# The IAEA remote and automated quality control methodology for radiography and mammography

**DOI:** 10.1002/acm2.13431

**Published:** 2021-10-08

**Authors:** Patricia Mora, Douglas Pfeiffer, Gouzhi Zhang, Hilde Bosmans, Harry Delis, Zahra Razi, Manuel Arreola, Virginia Tsapaki

**Affiliations:** ^1^ San José Costa Rica; ^2^ Boulder Community Health Boulder CO USA; ^3^ University Hospitals of the KU Leuven Belgium; ^4^ University of Patras Greece; ^5^ University of Florida Gainesville Gainesville FL USA; ^6^ Human Health Division International Atomic Energy Agency Vienna Austria

**Keywords:** automatic, detectability index, quality control, remote support

## Abstract

Radiography remains the most widely used imaging modality throughout the world. Additionally, while it has been demonstrated that a quality control (QC) program, especially in mammography, improves image quality, weekly technologist QC testing might be lacking even where there is clinical qualified medical physicist (CQMP) support. Therefore, the International Atomic Energy Agency (IAEA) developed simple QC phantoms that can easily be used on a regular basis (daily/weekly) for radiography and mammography. These are simple in design and use materials that are easily accessible in most parts of the world. A software application is also developed that automatically analyzes images and Digital Imaging and Communications in Medicine (DICOM) header information. It exports data to a comma‐separated values (CSV) file that is read by a Microsoft Excel® spreadsheet for documentation and graphical analysis. The phantom and the software were tested in four institutions (in Costa Rica and the United States of America) both on computed radiography and direct digital mammography and radiography systems. Data were collected over a 3‐year period. No corrective actions were taken on the data, but service was performed on two of the units. Results demonstrated noise that could be attributed to suboptimal placement of the phantom and incorrect data being put into the DICOM header. Preliminary evaluation of the IAEA methodology has demonstrated that it can provide meaningful QC data that are sensitive to changes in the imaging systems. Care must be taken at implementation to properly train personnel and ensure that the image data, including the DICOM header, are being correctly transmitted. The methodology gives the opportunity for a single CQMP to provide QC services even to remote sites where travel is prohibitive, and it is feasible and easy to implement.

## 1 INTRODUCTION

Radiography comprises the bulk of imaging performed across the world. Even with rapid development and deployment of advanced imaging modalities, such as computed tomography and magnetic resonance imaging, radiography remains central to patient care. Despite this, radiographic imaging systems receive some of the least technologist quality control (QC) efforts (e.g., weekly phantom imaging) of any imaging modality even though such regular QC testing is universally accepted as relevant. This remains true even in some facilities that have access to medical physics services, but is especially prevalent in underserved countries. The introduction of such programs is expected to have a positive impact on both reducing patient radiation exposure and improving image quality (IQ).^1^

Mammography is another important modality as its main purpose is to facilitate breast cancer detection at a point earlier in its natural progression than is possible by clinical examination. To detect breast cancer accurately and at the earliest possible stage, the image must have excellent contrast to reveal mass densities and fibrous structures radiating from them that are characteristic of cancer or appropriate spatial resolution to image calcifications, their number, and their shape.^2^, ^3^ This can only be realized when mammography systems perform accurately and safely. Effective quality assurance (QA) and QC programs have a positive impact on improving IQ and reducing patient exposure. QA is a framework to ensure that X‐ray facilities produce consistent, high‐quality images with minimum exposure to patients and personnel.^4^ QC is an essential part of QA that involves periodic and annual testing of all components of an imaging system.^4^

Per the IAEA, the professionals responsible for oversight of QA/QC programs of imaging equipment typically are the clinical qualified medical physicists (CQMP).^5^, ^6^ In many areas of the world, particularly in radiology, CQMP support is minimal or even nonexistent. This leaves many facilities with little or no guidance to implement a QA program in the imaging department. Under these conditions, imaging devices may go for their entire useful life without ever being tested, neither for regulatory compliance nor for radiation safety or IQ. Radiography and mammography modalities may never be evaluated on whether the clinical images they provide are of adequate diagnostic quality or not. Such a situation can lead to inadequate patient care and possibly excessive radiation exposure. While regulatory requirements may enforce annual performance evaluations in some countries, monitoring of the imaging equipment should not be limited to this infrequent testing, as this is not adequate to detect short‐term fluctuations or slow drift of some critical components of the imaging chain. Furthermore, many facilities have limited time to devote to QC, or in many instances, there is no designated person for this task, leading to different individuals performing the QC testing each time, thus, leading to inconsistency in evaluating the images. It was, therefore, necessary to develop a tool that is user‐independent and is straightforward in its application. Finally, traditional IQ metrics, such as the use of line pair patterns and the visibility of low contrast objects, are inherently subjective. Additionally, these measurements can be time consuming. This makes their use in a robust QA/QC program problematic or unreliable.

To help alleviate both the issue of lack of CQMP support in radiology and to ensure at least a minimal testing of radiographic equipment, the International Atomic Energy Agency (IAEA) developed a methodology for remote and automated QC incorporating simple phantoms and associated software and Microsoft Excel® spreadsheets that allow the CQMP to remotely supervise a QA/QC program for multiple department rooms or health facilities.^7^ The phantoms, while simple in design and inexpensive to fabricate, allow for sophisticated evaluation of radiographic and mammographic IQ. The software analysis tool developed is called Automated Tool for Image Analysis (ATIA) and allows for simple analysis either by the CQMP of the health facility or remotely by the CQMP supervising the QA program. Both simple and advanced metrics are evaluated using this software. The detectability index (*d*') that is calculated from the test images, could be used for quality improvement.^8^ The concept can be easily extended from conventional radiography to mammographic imaging.^9^, ^10^

The aim of this work was to implement and validate the new IAEA methodology and phantoms and software proposed were evaluated in terms of their functionality. The results of this pilot study are presented here.

## 2 MATERIALS AND METHODS

**FIGURE 1:**
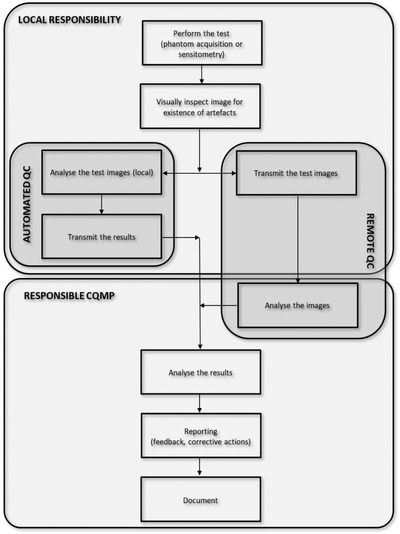
Basic concept of remote and automated QC as suggested in the IAEA methodology. Images are acquired and approved at the health facility. If the capability exists, these images are then sent to a central location for analysis by the supervising CQMP. This may be accomplished automatically in a more sophisticated system or directly by the CQMP. Data is logged and inspected for elements being outside of control limits or negative trends

Ideally, for every new system, the CQMP should perform a commissioning test protocol. To ensure a proper balance of radiation dose and IQ, it is essential that the X‐ray systems are well calibrated and optimized. This must be verified by the CQMP. During the commissioning test, operating levels and control limits of the X‐ray system can be set. Following the baseline tests, tracking of X‐ray system performance over time is evaluated by means of simple QC tests as described in the IAEA remote and automated QC methodology.^7^ The methodology consists of different components and responsibilities as shown in Figure 1. Detailed description of phantoms, software, and proposed analysis can be found below.

### 2.1 Design of the phantom

**FIGURE 2:**
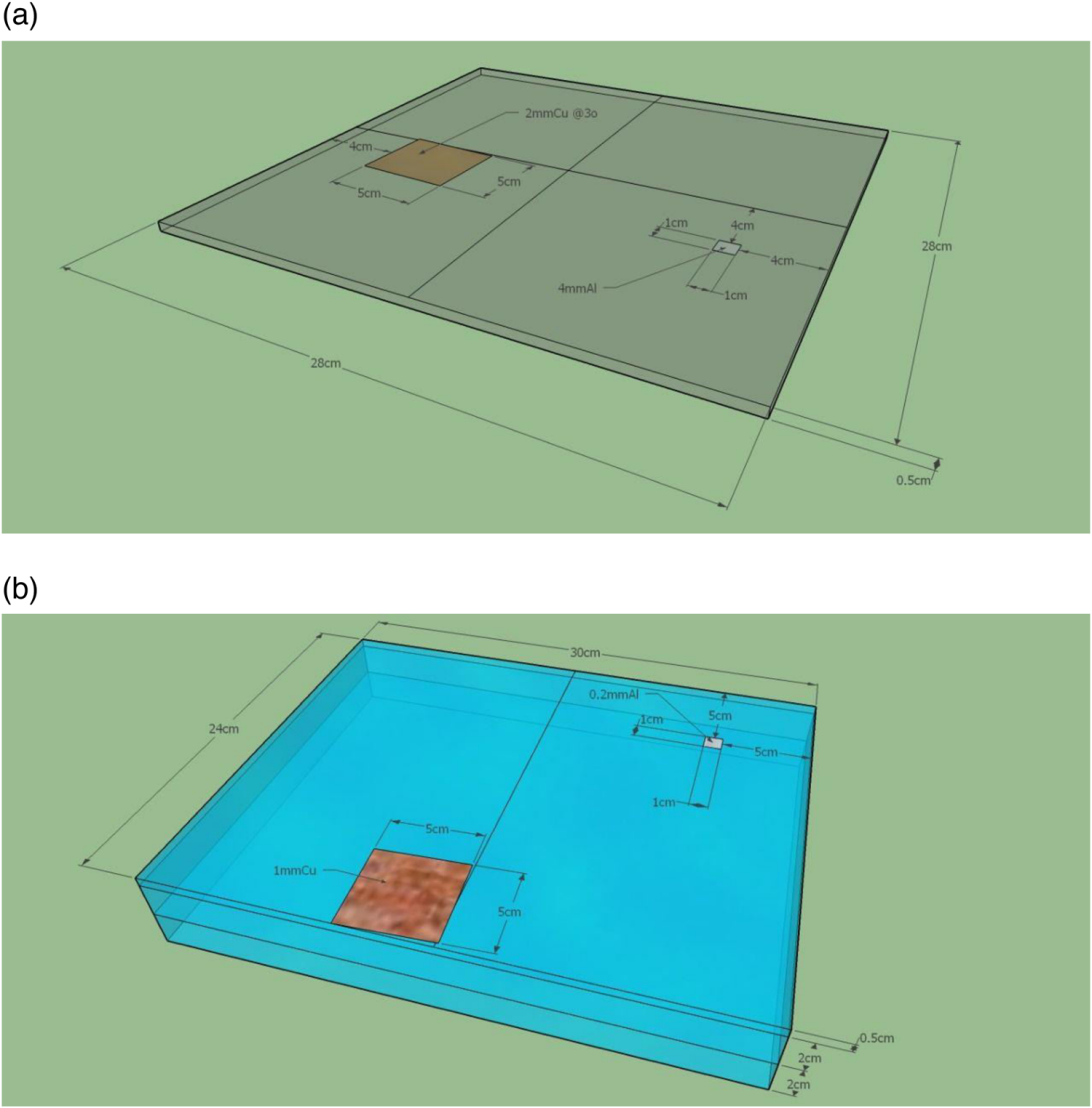
(a) Radiographic phantom as proposed in the IAEA methodology is presented. Note that only the target plate is shown for the radiographic phantom; the copper attenuator is not shown. It simply consists of a 5 mm thick sheet of PMMA with a copper square and an aluminum square affixed to it as shown, (b) The mammography phantom as proposed in the IAEA methodology is shown. In this phantom, 4 cm is used as the main attenuator. The 5 mm thick target plate has a copper and an aluminum square affixed to it

The phantoms proposed are simple and relatively inexpensive, as they use materials which can be purchased and manufactured locally. The phantom for general radiography testing generates a spectrum that is representative of a patient by means of a 0.2 cm thick homogenous copper (Cu) plate. If it is more cost effective, this sheet may be composed of several thinner sheets stacked together totaling 0.2 cm, such as two sheets each 0.1 cm thick. The second part of the phantom consists of a target plate of poly methyl methacrylate (PMMA) that is 28 × 28 cm and 0.5 cm thick. Two rectangular inserts are placed on this piece as shown in (Figure 2a). The first target is a 5 × 5 cm Cu square of 0.2 cm thickness. The Cu piece is used for modulation transfer function (MTF) and detectability index (*d*') analysis. Therefore, it is critical that the Cu square rests flat on the PMMA target plate and is angled 2–5° from the edge of the target plate (and therefore angled 2–5^o^ from the digital image matrix) for accurate MTF determinations. While the specific angle of the Cu square is not critical, it is suggested to use a protractor to ensure that angle is within the specified range. The edges of this object should be as smooth as possible. The second target is a 1 × 1 cm square of aluminum (Al), 0.4 cm thick. This is used for contrast‐to‐noise ratio, signal‐difference‐to‐noise ratio (SDNR), and detectability index (*d'*) analysis (Figure 2a).

The mammography phantom is of a very similar design. In this case, the uniform attenuator is a 24 × 30 × 4 cm slab of PMMA. As before, it may be more cost effective to stack together thinner slabs to total 4 cm. The target plate is 24 × 30 cm by 0.5 cm PMMA. The Cu square for MTF determination is 0.1 cm thick and the Al square is 0.02 cm thick (Figure 2b).

To help control cost, the components of the phantom do not have specific tolerances associated with them. Due to the simple design of the phantoms, it is expected that facilities should be able to fabricate them in‐house.

While many phantoms are currently produced for both mammographic and radiographic QC, such as the American College of Radiology Mammography Accreditation Program phantom or The Radiography Fluoroscopy QA Phantom (CIRS Inc., Norfolk, VA, USA) they differ from the proposed phantoms in several respects. The most important difference is the cost of the phantom. Commercial phantoms can cost hundreds, if not thousands, of dollars, which puts them out of reach for many imaging centers in developing countries. The proposed phantom is intended to be constructed in‐house with commonly available materials. Secondly, most phantoms available are subjective in nature. Some, such as the CDMAM phantom (Artinis, Nijmegen, Netherlands), have analysis software available, this software may add additional cost. The analysis software and spreadsheets for the proposed phantom are freely available from the IAEA and provide objective results.

Commercial phantoms have the advantage of being very reproducible in construction. It is acknowledged that the proposed phantoms will likely demonstrate large variability in construction, they are not intended for intercomparison or standard setting, so the variability will not pose an issue.

### 2.2 Imaging acquisition

The procedure assumes that the imaging system under consideration possesses the ability to generate and transfer unprocessed (i.e., “for processing”) QC images. In these images, only basic dead pixel, flat field, and similar implicit correction algorithms have been applied, but no frequency‐based or look‐up table mapping of any kind has been used. If “for processing” images are not available, then “for presentation” (processed) images could be used, though minimal processing should be applied.

The phantoms should be exposed with clinically relevant parameters that embrace as many imaging components as possible (tube, collimator, grid, detector, automatic exposure control [AEC]). For the radiographic phantom, technical settings for a standard, medium‐sized abdomen protocol are to be used, such as 100 cm source to image distance (SID), 80 kilovolts peak (kVp), and AEC or 10 mAs. For the mammography phantom, either a fully automatic parameter setting or 28 kVp with semiautomatic AEC can be used. If not recorded in the DICOM header, the resultant exposure parameters (e.g., anode/filter, kVp, and tube load [mAs]) must be recorded for stability tracking. For radiography, the X‐ray field must be collimated to the test plate to minimize extraneous scatter.

When imaging the phantom, the following items are also essential:

- The phantom must be positioned correctly, with particular attention to ensuring that the phantom is not rotated relative to the edge of the radiation field.
- The same kVp (for example, 80 kVp for radiographic systems and 28 kVp for mammographic systems) must be used every time, unless automatic controls have been employed.
- The radiation field must be collimated to include the entire phantom and should be consistent from exposure to exposure.
- For Computed Radiography (CR) systems, a test cassette must be designated and labeled (which may be used clinically as well) and used each time.
- Two‐detector Digital Radiography (DR) systems with an upright bucky detector and a table bucky detector require that test images for each of the detectors be acquired. For systems with a single detector that is used at both buckys, it is advisable to test at both to ensure that the AEC is working properly at both.
- The same exam and view selection must be made every time (e.g., Anteroposterior [AP] abdomen, medium adult).
- The same image processing selections must be chosen every time (e.g., flat field, QC, unprocessed).

To achieve these goals, adequate training of the technologists who will be acquiring the images is essential. This is one of the tasks that must be performed by the overseeing QCMP at the initiation of the program.

### 2.3 ATIA software tool

In the IAEA methodology and using the ATIA software tool, subjective IQ evaluations are replaced by quantitative, advanced metrics, such as SDNR, MTF, and detectability index (d'). These metrics are calculated by the ATIA software application. None of these metrics depend on the observer, so the impact of different individuals performing the analysis is negligible, except for consistent placement of the phantom.

ATIA is a standalone and portable application that has been developed to facilitate the analysis of images and the determination of quality metrics on acquired QC images produced by the two phantoms described above. This tool was developed in C/C++. The DCMTK library (OFFIS, Germany) was used for handling the DICOM data structures, the FFTW library^11^ for the fast Fourier transform, the GNU scientific library^12^ for part of the numerical computations, and the Qt (Digia Plc.) for the graphical interface. ATIA runs with current Microsoft Windows and Apple macOS systems.

In a broad sense, the MTF expresses the ability of a system to image fine details. While multiple methods for determining the MTF exist, one of the most robust and easiest to implement automatically is to use the Fourier transform of an image of a sharp edge.^13^, ^14^, ^15^, ^16^ The Fourier transform of the edge yields an edge spread function. The derivative of the edge spread function yields a line spread function. Finally, the inverse Fourier transform of the line spread function yields the MTF. The Cu square in the described phantoms is positioned and used for this purpose.

Contrast is the ability of a system to discern an object with a small signal difference from the background. An object with a smaller signal difference is more difficult to see than one with a larger signal difference. Furthermore, greater noise in the background will make it harder to visualize an object with a given signal difference. This task is often described by the SDNR, which is given by

(1) $$SDNR\;=\;\frac{S_{background}\;-\;S_{target}}{\sigma_{background}},$$

where *S*_x_ = mean signal in the ROIs and σ_background_ = background noise. In the two phantoms, the small Al squares are used for SDNR determination. The ROIs (5 × 5 mm) are automatically sized and placed by the analysis software.

The Normalized Noise Power Spectrum (NNPS) is estimated from a square of 512 × 512 pixels in a homogeneous area of the phantom image. A total of 3 × 3 half‐overlapping ROIs, each with 256 × 256 pixels, are used for the 2‐dimensional (2D) NNPS calculation. To remove the large‐scale gradient, the large area is flattened with linear fitting consecutively across the two orthogonal directions. The NNPS is then calculated using a standard formula.^17^, ^18^

An image was simulated with a Gaussian pixel profile and added Poisson noise. The NNPS was calculated both with and without detrending the Gaussian profile. Detrending decreases the NNPS at the lowest frequencies, but frequencies above the Nyquist are not affected. It is often preferable to filter the lower frequencies, which are typically due to the X‐ray tube, filter, or beam, as it is the detector characteristics that are of interest.^19^

The presampled MTF is measured from the edges of the Cu plate in the phantom.^20^ The plate is placed near the center of the detector and rotated slightly to give an angle between 2° and 5° with respect to the pixel matrix. Directional MTF is obtained at highly supersampled pseudofrequencies as created by the slanted horizontal and vertical edge. Then the two orthogonal MTF curves are averaged and evaluated at the same frequencies as the NNPS.

Even though the SDNR, MTF and NNPS remove subjectivity from the analysis, they still suffer from the fact that their clinical relevance is limited. They grossly simplify the challenges of interpreting diagnostic radiologic images. To help overcome this, a newer metric has been developed, known as the detectability index (*d*'). This index relates subjective measurements of contrast, NNPS and MTF to actual, clinical interpretation tasks. The d*'* for a Non‐Prewhitening Model Observer with Eye Filter (NPWE) is determined^9^, ^14^:

(2) $$d^{\prime }=\;\frac{\sqrt{2\pi }C\int_{0}^{\infty }S^{2}\left(u\right)MTF^{2}\left(u\right)\;VTF^{2}\left(u\right)u\;du}{\sqrt{\int_{0}^{\infty }S^{2}\left(u\right)MTF^{2}\left(u\right)\;VTF^{4}\left(u\right)NNPS\left(u\right)u\;du}},$$

where *u* represents the frequency, *C* is the nominal contrast of the object, *S* is the object shape function defined by Fourier transform of a disk with a diameter *D* = 0.3, 4.0 mm for radiography and *D* = 0.1, 0.25 mm for mammography, VTF is the visual transfer function defined with a viewing distance of 400 mm.^15^, ^16^ The viewing distance must be defined in the VTF due to the dependence on object size and the angle it subtends to the eye. In this calculation, the MTF and NNPS of the orthogonal directions are averaged and then evaluated at the same frequencies via interpolation.

The results of the ATIA analysis are exported into a CSV file. A Microsoft Excel® spreadsheet has been developed for compilation, plotting, and acceptability determination of the data. The CSV file is read by the spreadsheet and data are extracted. After baselines have been established and action levels have been put into place, the extracted data are added to the database, plotted, and can be compared to action limits.

Artifacts or nonuniformities in the signal can make an otherwise excellent image useless. These problems can occur suddenly and may have to be remediated. It is, therefore, essential to include artefact and uniformity analysis in any QC program and to give local personnel the tools to read the image or send data for advice to a remote center. The ATIA application includes a function for highlighting areas of nonuniformity and artifacts. This function may be run on the image with the target plate, recognizing that the test targets will be identified by the application, or it can be run on a separate, uniform image with only the base attenuator. In either case, images should be visually reviewed by either the facility or the CQMP to ensure that artifacts and nonuniformities cannot hide any pathological condition in the patient. The variance map is an analysis of the variation in pixel values throughout the image. It is calculated by evaluating the local variance across the entire image area with a kernel size of 2 × 2 mm and normalized with respect to the variance found in the large area that is used for evaluating the NNPS. For easy observation, the map is color coded in scale from green (minimum) to red (maximum) and exported in common photographic format, where the range is set by the user. Potentially problematic locations on the image can be quickly spotted, as nonvarying areas will appear to be green while abrupt changes will be red.

### 2.4 Image analysis using ATIA software

**FIGURE 3:**
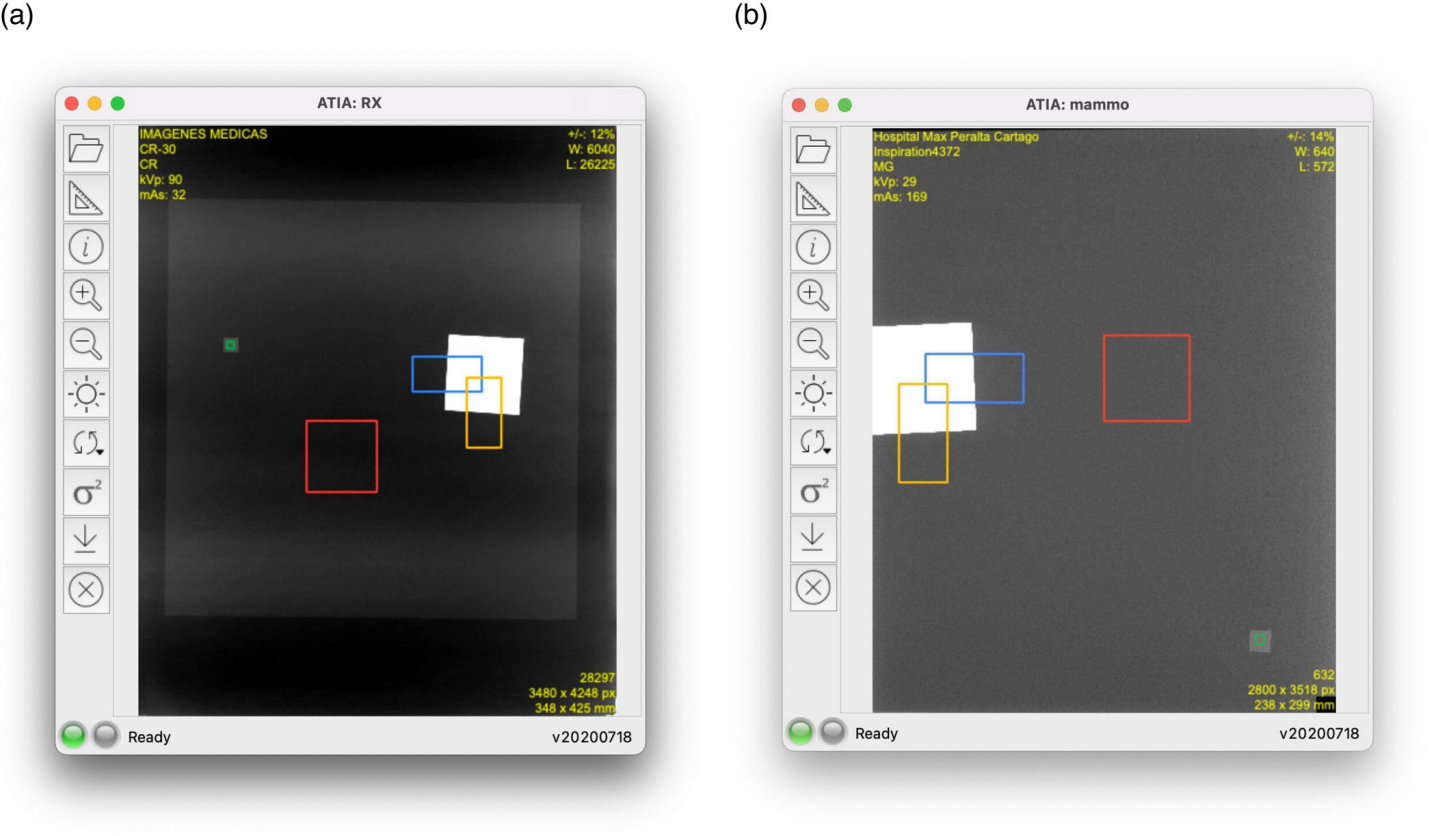
(a) ATIA interface for the radiography phantom. The ROIs used by ATIA have been automatically identified. (b) ATIA interface for the mammography phantom. The ROIs used by ATIA have been automatically identified.

Figure 3a and b shows the ATIA interface for both types of phantom images. The first step is to select or drag the QC image into the display panel, upon which ATIA begins an initialization procedure to automatically locate the ROIs and place the indicators to the best of its feature‐recognizing ability. Such initialization takes only a few seconds and should always work if the phantom and acquisition follow the IAEA methodology. In the rare case that the ROI placement algorithm fails, the ROIs can be manually dragged to the proper location. The display panel supports zooming, panning, and windowing operations on the image view. Once ROIs are all set, the measurements and calculations are performed by clicking on the measurement button. ATIA then provides the following IQ metrics: SDNR, SNR, MTF, NNPS, and detectability index (*d*'). The user has the option to export all the metrics as well as a group of selected information tags from the DICOM header in plain text format, or as a CSV file. A Microsoft Excel® worksheet with built‐in macros produces control charts for mAs, kVp, organ dose, entrance dose, exposure index, SNR, SDNR, MTF (horizontal and vertical characteristic frequencies at 50%, 20%, and 10%), and detectability index (*d*'). ATIA also exports the variance map.

### 2.5 Remote QC

Remote QC relies on an automated analysis of an entire image rather than using localized simple measurements made manually on the image. It consists of the following major components: local image acquisition, local image verification and artefact analysis, image upload, centralized image analysis, result analysis reporting, and feedback. The images are acquired by local personnel, such as CQMPs or medical radiological technologists, who must be trained in uploading the images into the centralized system. The server may or may not be located at the facility. Advanced processing of the uploaded images is performed centrally by the CQMP using ATIA to extract quantitative indices related to noise, uniformity, and artefact detection. Clearly, a system must be in place to generate immediate feedback routed to the facility and the supervising CQMP regarding any inadequate performance of the system and the need for follow‐up or corrective actions. Local QC, as opposed to remote QC, consists of the following major components: local data acquisition, local image verification and artefact analysis, local automated image analysis, data upload, centralized results analysis, and reporting and feedback. The measurements required for the automated QC are meant to be performed either automatically or by the local personnel, who are expected to be trained in performing the measurements and entering the data into the centralized system, which may not necessarily be located at the facility. Daily or weekly measurements do not require the onsite presence of the CQMP on a regular basis. Data can be uploaded for centralized analysis and reviewed by a CQMP.

### 2.6 Verification of IAEA methodology (Pilot survey in test institutions)

The IAEA methodology was implemented under a pilot study in Costa Rica and the USA starting the summer of 2017. A total of four medical centers participated in this prospective study, two in Costa Rica and two in the USA. One CR and seven DR X‐ray radiography units were evaluated, whereas for mammography, two DR units were evaluated. In Table 1, the period of data collection and number of images obtained is shown.

**TABLE 1:** Information on participating centers, equipment evaluated, phantom used, data collection period, and total images recollected are shown

Country	Facility	X‐ray unit technology	Model	Period of data collection	Major changes or variables	Number of images
Costa Rica	CR1	CR Radiology	AGFA CR‐30	July 2017–April 2020	X‐ray tube changed	83
	CR2	DR Mammography	Siemens Inspiration	July2017–March 2020	Detector changed and autosegmentation were initially turned on and later turned off	78
USA	USA1	DR Radiology	AGFA CR_10X retrofit with DX‐D 100 DR	June 2018–July 2019	Data for X‐ray field collimated to phantom and open to detector size	89
	USA1	DR Radiology	AGFA CR_10X retrofit with DX‐D 100 DR	June 2018–July 2019		89
	USA1	DR Radiology	Siemens Multix Select	April 2018–July 2019		63
	USA1	DR Radiology	Siemens Multix Select	March 218–July 2019		55
	USA1	DR Radiology	Carestream DRX‐Revolution Mobile	June 2018–July 2019	Data for 2 SIDs and 2 imaging acquisition protocol (including pattern and abdomen)	86
	USA1	DR Radiology	Carestream DRX‐Revolution Mobile	June 2018–July 2019		86
	USA2	DR Mammography	GE Essential	Jul 2017–Oct 2017		18
	USA2	DR Radiology	Siemens	June 2018		16

The two centers from Costa Rica are still collecting data on a weekly basis. Note that “pattern” on the Carestream systems produces an image with no processing applied—effectively a “for processing” image.

Both local and remote control of data have been developed and tested, with the two centers in the USA analyzing the images locally and transmitting the results to the project team, while centers in Costa Rica directly transmitted the test images. Image data were collected daily at the beginning to establish baseline values for all metrics and then later on a weekly basis. The format for all images was unprocessed (i.e., with the DICOM tag “for processing”).

The fluctuations in the results related to phantom positioning and data analysis were interrogated by imaging each phantom ten times with a small move or rotation between each exposure to mimic the normal variation in positioning of the phantom. The phantom was also imaged ten times with no movement between the exposures to characterize the inherent variability. Analysis of the same image five times demonstrated no variability in the analysis itself.

During the evaluation process, no effort was made to take corrective measures based on the data.

## 3 RESULTS

The coefficient of variation (CV) of the different metrics both due to variation in positioning and due to inherent variability are shown in Table 2a and 2b. The mammographic phantom is relatively insensitive to movement with the largest CV equal to 8.9% for the SDNR measurements. However, SDNR and SNR remained more stable with the phantom positioned in a consistent location. Conversely, the radiographic phantom data are fluctuating more, with CV between 17% for SNR and 57% for the line pairs, at MTF 10% values, respectively. Interestingly, even with the larger CVs for the other descriptors, detectability index (*d*'), the primary IQ descriptor, remained relatively invariant with CV (4.8% and 8.2% for the 0.3 and 4.0 mm targets, respectively). The inherent variation demonstrated similar trends with smaller magnitudes, there being little variation in the mammographic phantom, the largest CV equal to 1.6 %. The radiographic phantom showed greater variability, with the greatest CV being 25.3% for the 10% MTF in the vertical direction. Again, detectability index (*d*') demonstrated low variability with CVs of 1.7% and 1.8% for 0.3 and 4 mm targets, respectively.

**TABLE 2a:** Reproducibility of the mammographic phantom (with and without movement)

Reproducibility of the mammographic phantom										
			Vertical MTF			Horizontal MTF			*d*'(mm)	
	SDNR	SNR	50%	20%	10%	50%	20%	10%	*D* = 0.1	*D* = 0.25
CV% with movement	8.9	6.5	1	1.8	2.9	2	2.6	3.7	1.4	1.4
CV% without movement	1.3	1.1	2.6	0.9	1.7	1.7	1.6	1.4	1.5	1.6

The movement consisted of small shifts in the position of the phantom to mimic the displacement likely to occur during the weekly QC testing. It can be seen that SDNR and SNR are more sensitive to displacement that are the other indexes.

**TABLE 2b:** Reproducibility of the radiographic phantom (with and without movement)

Reproducibility of the radiographic phantom										
			Vertical MTF			Horizontal MTF			*d*' (mm)	
	SDNR	SNR	50%	20%	10%	50%	20%	10%	*D* = 0.3	*D* = 4
CV% with movement	17	17	41	26	33	32	32	57	4.8	8.2
CV% without movement	1.3	1.4	5.5	8.4	6.2	4.3	11.6	25.3	1.7	1.76

This phantom is more sensitive to movement, so careful placement of the target plate by the QC technologist is essential.

**FIGURE 4:**
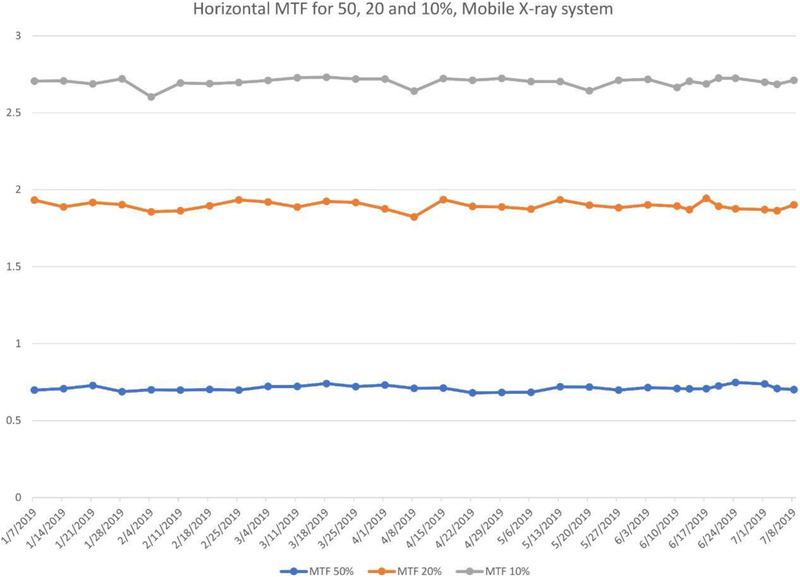
Tracking of the line pairs at MTF values (a) 50%, (b) 20% and (c) 10% for a Carestream DRX with Revolution Mobile X‐ray System. MTF is seen to be stable on this unit

Once the weekly data from ATIA were uploaded into the Excel control charts, all parameters were followed over time to track their behavior. Metrics studied with this methodology can be used to verify the consistency of the equipment, detect changes, and predict the need to repair or upgrade equipment. An overview of all the graphs is displayed on the first page of the Excel workbook so the user can rapidly detect any abnormalities. On subsequent pages, each parameter is individually displayed for a more comprehensive analysis. For each metric, the user can set the upper and lower limits to 10, 15%, or two standard deviations (SD). Figure 4 is the tracking of 50, 20, and 10% MTF frequencies for a DR X‐ray unit. MTF values are stable over time.

**FIGURE 5:**
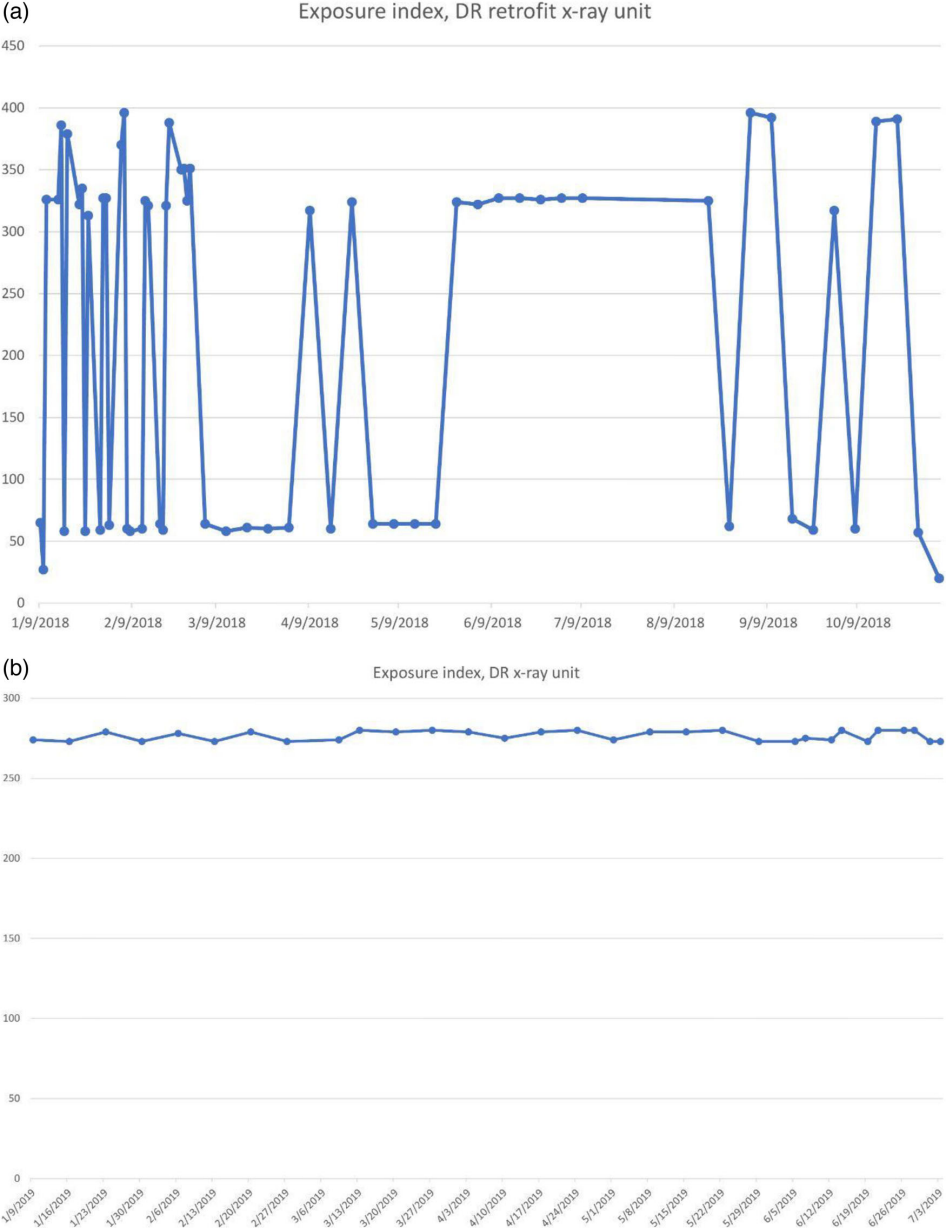
Fluctuation of Exposure Index for (a) an Agfa CR‐10X system and (b) a Siemens Multix Select DR system. The large fluctuations in (a) are likely due to incorrect information being entered into the DICOM header

**FIGURE 6:**
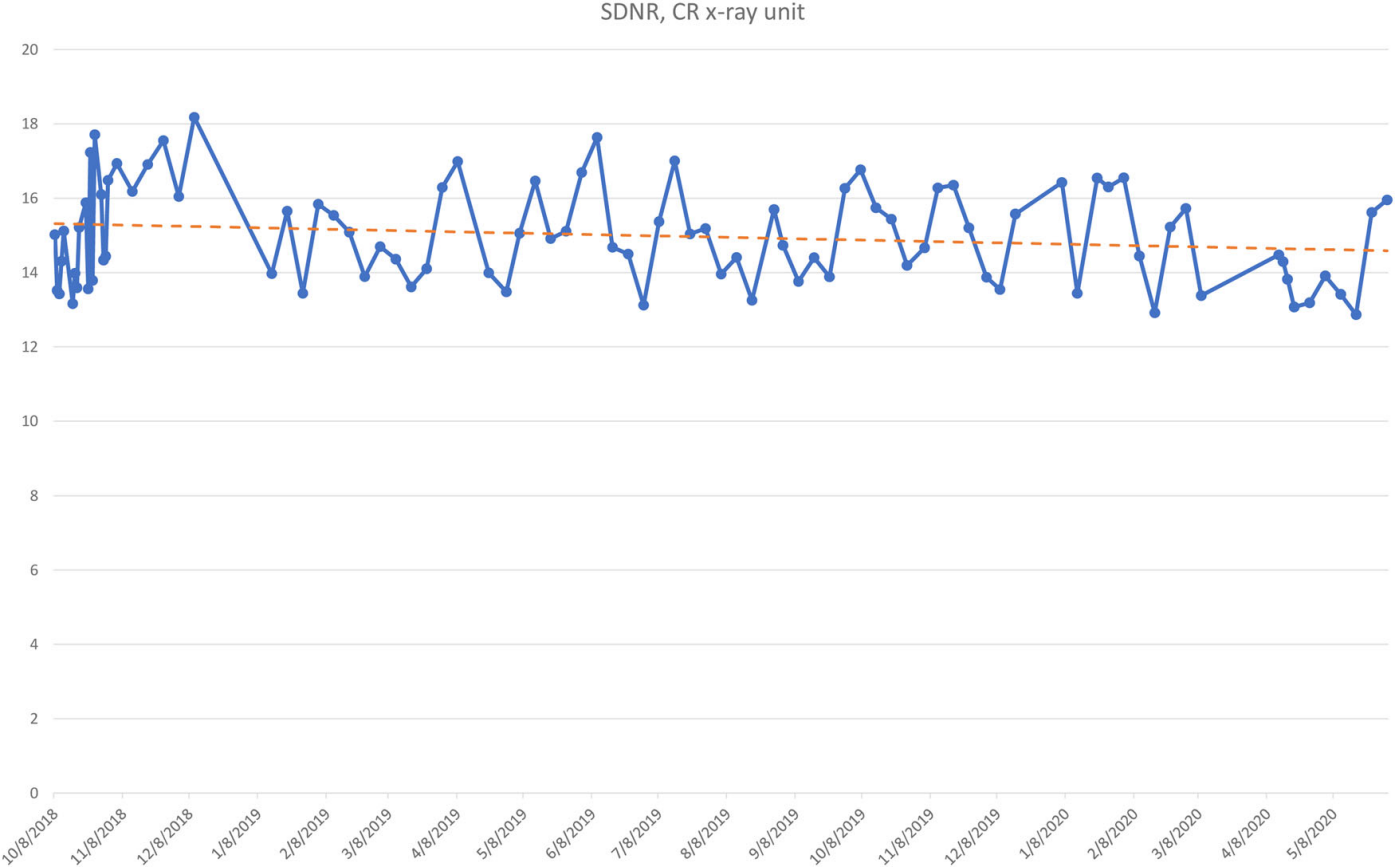
Long period of tracking (almost 2 years) for SDNR in an Agfa CR‐30 unit. While the SDNR is noisy, as noted above, it can be seen that the index is slowly dropping, indicating an issue that needs to be addressed

**FIGURE 7:**
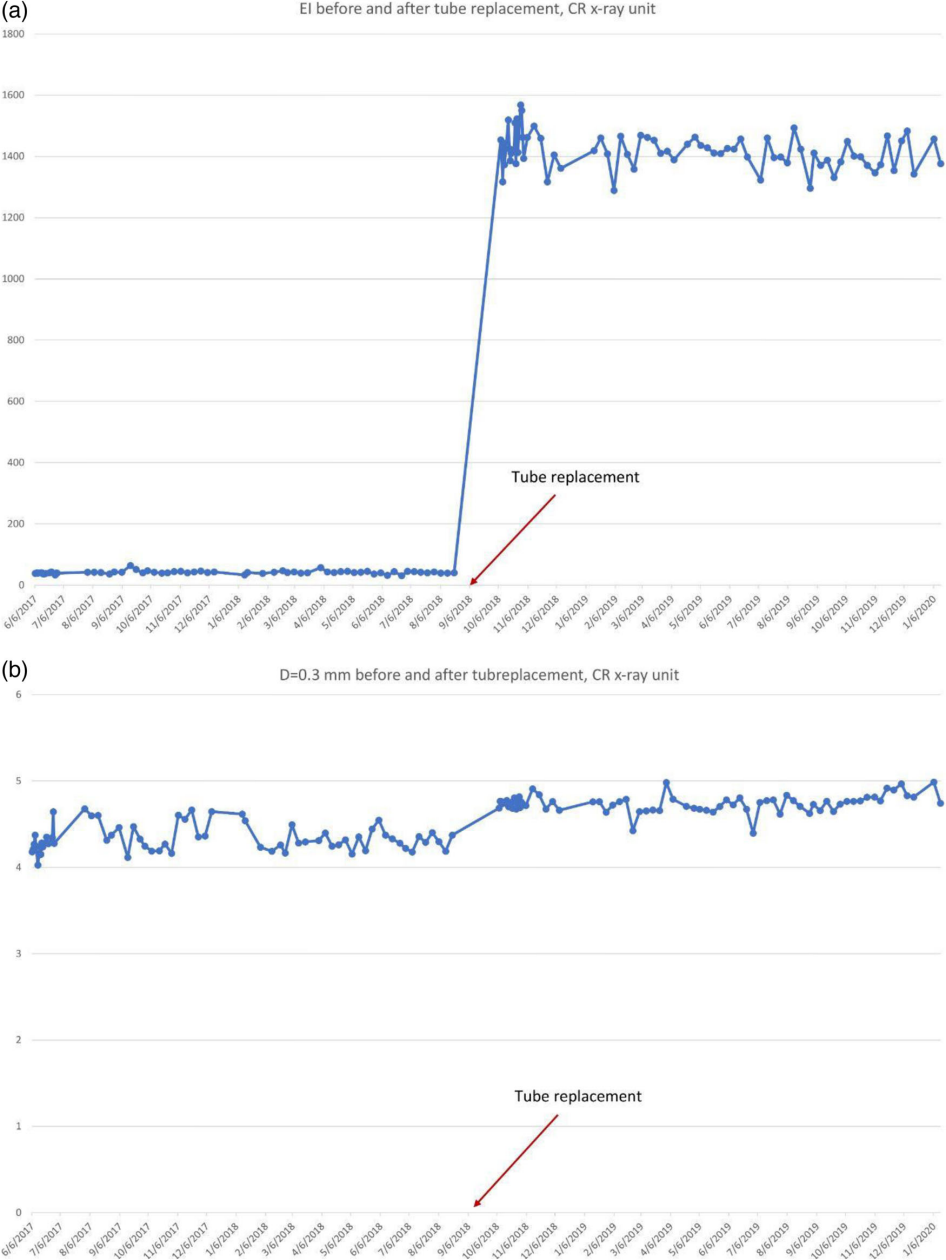
Change of X‐ray tube in October 2018, demonstrating in (a) an unexpectedly low value in EI changing to a clinically reasonable value, contemporaneous with recalibration of the EI. While not an intended aspect of this program, it can also reveal issues not associated with image quality. (b) A change in detectability index (d') (for 0.3mm disk) at installation of the new tube is noted

**FIGURE 8:**
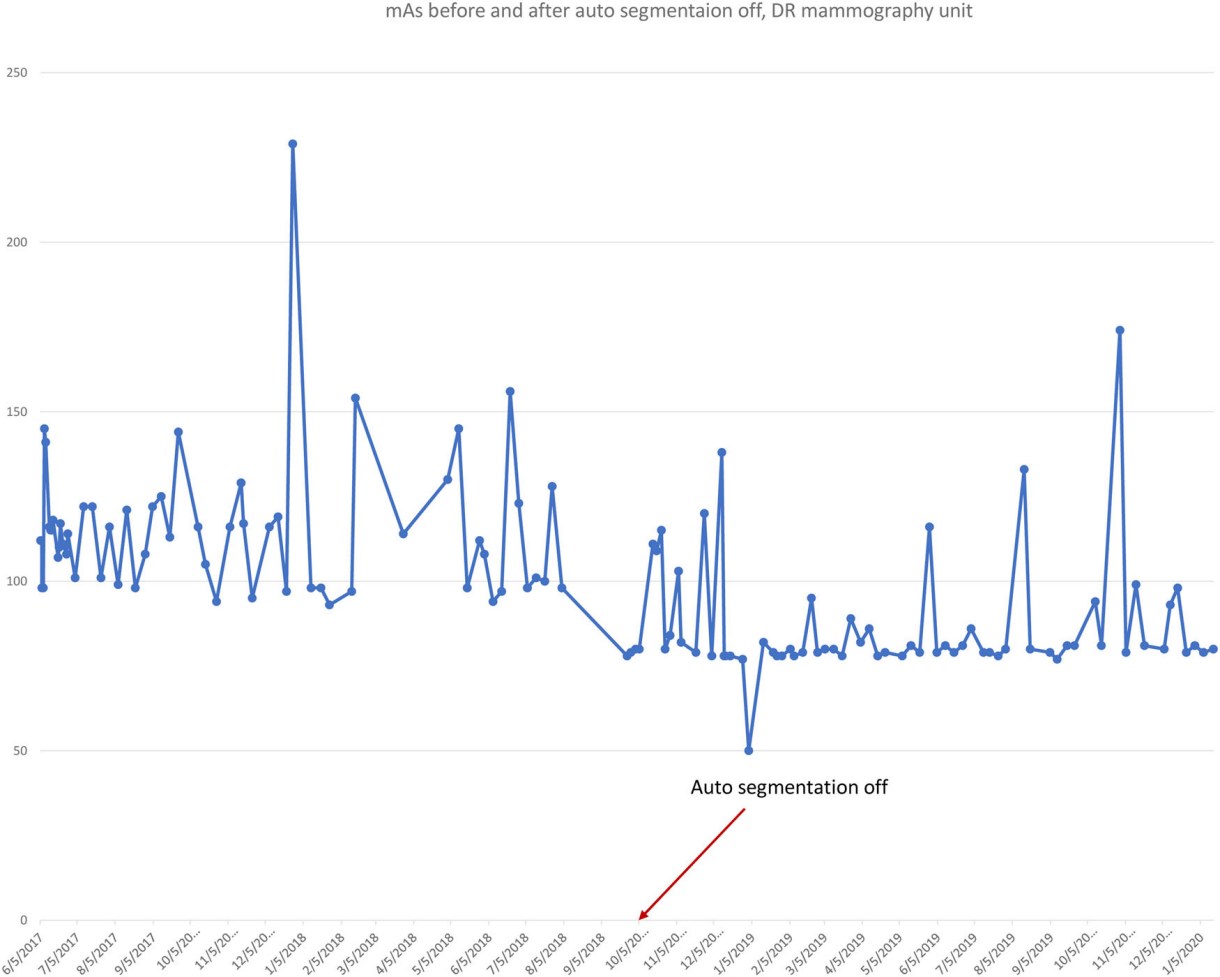
Tracking of mAs values of a mammography unit over a 3‐year period. In the first part of the graph, auto‐segmentation was “on” showing a large weekly variation on mAs. With auto‐segmentation “off”, starting October 2018 the mAs is more stable (standard deviation changed from 21.63 to 17.81), though still noisy. The cause of this variation was not investigated during this trial phase

**FIGURE 9:**
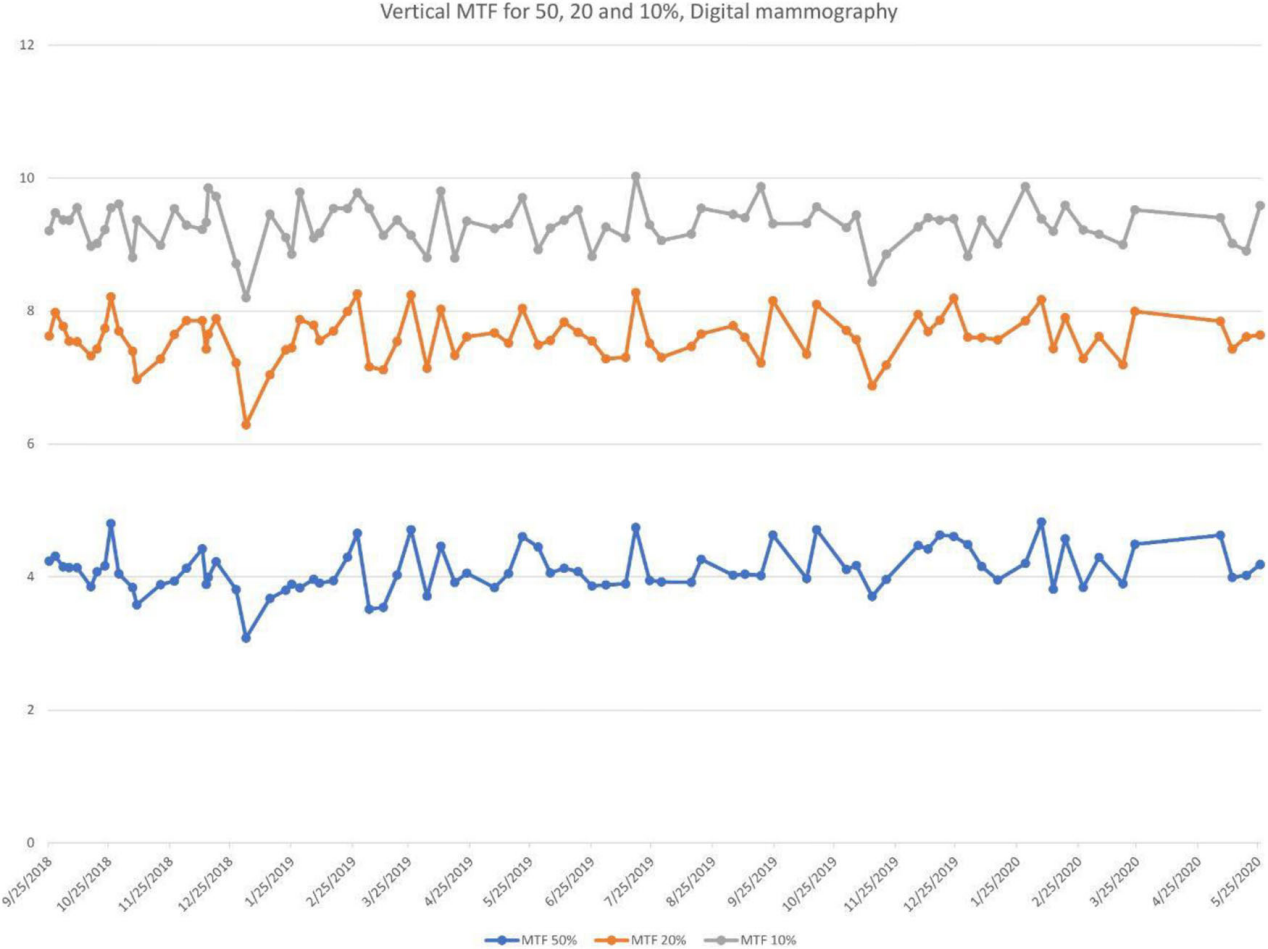
Line pairs at MTF at (a) 50%, (b) 20%, and (c) 10% on the vertical axis for Siemens Inspiration mammography unit. Unlike SDNR shown earlier, the MTF has remained relatively stable over time

**FIGURE 10:**
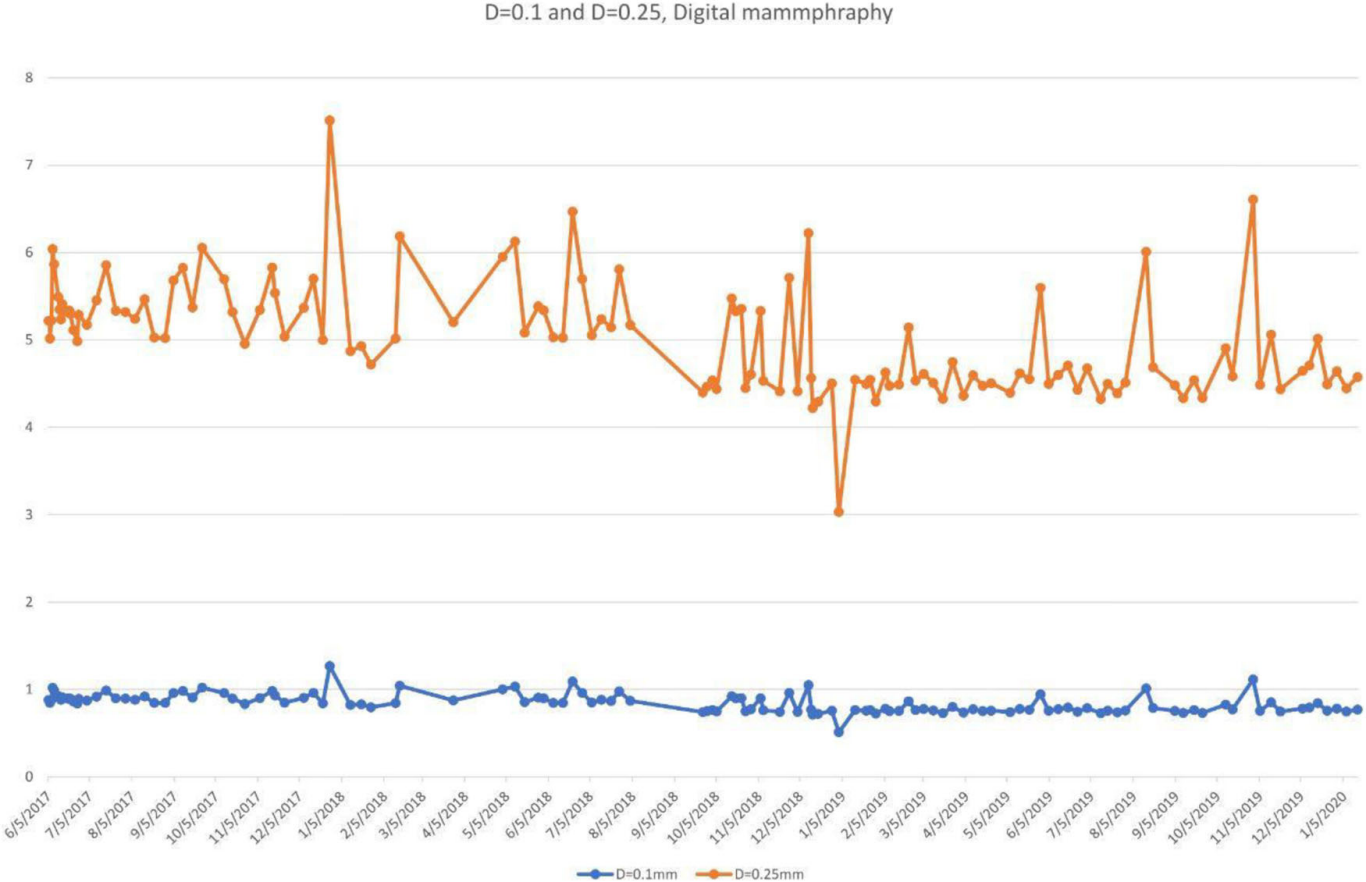
Detectability index (d') values for disks with diameter with diameter (a) 0.1 mm, and (b) 0.25 mm from a Siemens Inspiration mammography unit. The cause of the noise in these charts was not investigated during this trial phase.

**FIGURE 11:**
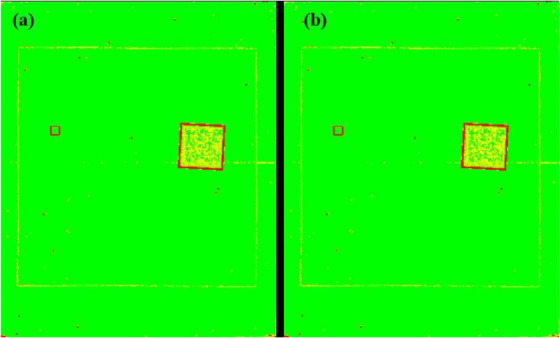
Variance maps of AGFA CR‐30 (a) before, and (b) after 23 months showing the same IP flaws in the same location (red dots)

**FIGURE 12:**
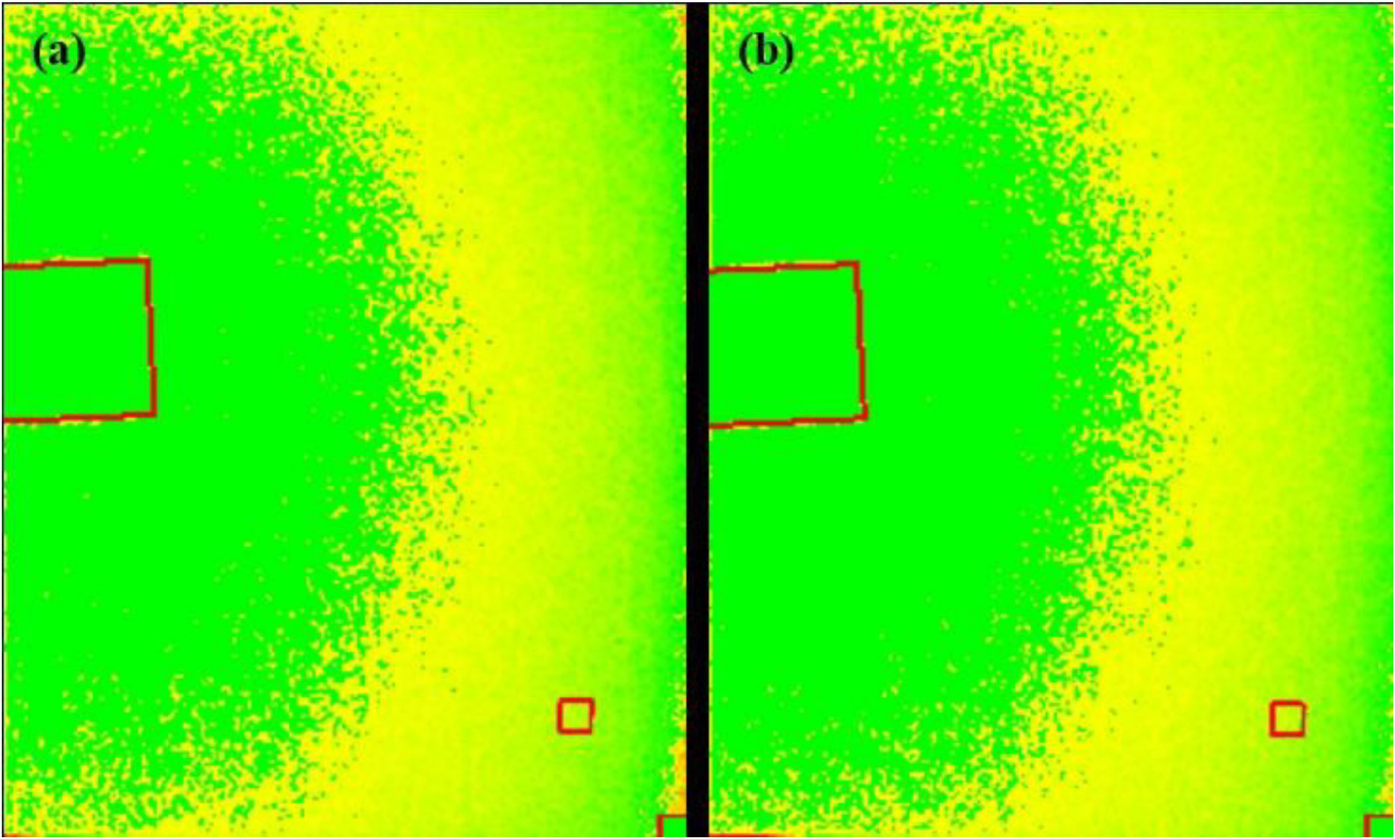
Variance map of mammography unit (a) before, and (b) after the detector was changed, see lower right corner in both images for deterioration

While most of the graphs showed that the metrics were compatible with stable devices, a few exceptions were observed:

1. The plot of exposure indexes (EIs) for a DR system (Figure 5a) demonstrates unusual behavior of alternating between two sets of values. It is clear that the lower values are erroneous, as such a low EI would result in unacceptable IQ. The values between 350 and 400 are clinically more reasonable. Such behavior must be investigated and corrected and is most probably due to errors in the procedure or in the information that is stored in the DICOM header. Other DR EIs from the same institution (Figure 5b), are reasonable and consistent.
2. Figure 6 shows the SDNR values over time, with a gradual decrease around June 2020. Intervention was planned to avoid allowing the system to degrade further and produce suboptimal images.
3. During implementation, one radiographic unit, incorporating an Agfa CR‐10X detector, had its X‐ray tube changed. In (Figure 7a), the initial EI control chart can be observed with values around 40 units; after the change, the EI raised to around 1400 units. Most likely, a different value was used to populate the EI DICOM tag following the service. It must have been recognized that 40 is an unusually low value and was likely not correct. Additionally, in Figure 7b the impact of a new tube on detectability index (*d*') (0.3 mm) on a CR system can also be appreciated. The unit in question does not have an AEC system. The output of the new tube is higher than the old tube. So, with no change in the manual techniques used, the detector is receiving more radiation, which improves the detectability index. Additionally, while focal spot size was not measured after installation of the X‐ray tube, it is possible that the new tube improved the MTF of the system.
4. Additionally, in Figure 7b the impact of a new tube on detectability index (*d*') (0.3 mm) can also be appreciated. It is promising to see that detectability increases with a new X‐ray tube, which may help to justify such an investment.
5. One mammographic unit has been followed for almost 3 years (starting in June 2017). Many of the initial fluctuations were due to the learning curve of the technologists involved with the test procedure: this X‐ray unit is located in a large hospital with many personnel rotating across various rooms. Even though training of all technologists was implemented, only a few of them followed the protocol correctly. Starting in October 2018, the autosegmentation was turned off to decrease fluctuations in the parameters, especially on mAs values. Figure 8 demonstrates the entire timeline of data for this unit. As can be noted, less fluctuations were registered after the autosegmentation was turned off (second half of data). This equipment has a very heavy workload and controlling external factors that might impact results was a challenge. For the same mammography unit, the line pairs at MTF 50, 20, and 10% are presented in Figure 9 and Figure 10a and b demonstrate detectability index (*d'*) for 0.1 and 0.25 mm, respectively. These stable results show the feasibility of detectability index (*d*') measurements at weekly QC.
6. Every week variance maps of the phantoms were also extracted from the ATIA software to check the homogeneity of images and the presence of eventual artefacts. Figure 11 shows variance maps for a CR cassette separated by 23 months (localization of dead pixels in the same location for the same IP cassette used) and Figure 12 shows variance maps for the mammography unit before and after detector change. Improvement in detector quality can be appreciated in the lower right‐hand corner.

## 4 DISCUSSION

The combined phantom and software procedure have been successfully implemented in four centers for 10 imaging devices. Equipment tracking with these tools has now run for more than 3 years and provided interesting and relevant findings. Overall, the measurements are sufficiently reliable to track the performance of the devices. A limitation of the study is that there is no proof that all significant technical problems with the devices have been detected as there was no alternative daily QC procedure implemented. It must be noted that there were no severe complaints reported by the clinicians about IQ associated with any of the equipment used in this study.

It must be realized that the proposed IAEA remote and automated QC methodology is not intended to undermine the importance of (or replace) regular testing by a CQMP. The methodology reveals the importance of daily/weekly QC testing in the everyday clinical routine as poor‐quality X‐ray images may hamper clinical care. The current phantom and software procedure are designed such that CQMP support is central to its success. Just as mammographic QC programs in Europe and USA have not supplanted regular CQMP testing, neither should this program supplant regular CQMP testing. It is essential to note that the phantoms should undergo the process of commissioning by a CQMP, on each specific system that is part of the program, to ensure proper utilization, to provide the required training to the staff, and to establish baseline values. The test tools that have been described make use of common materials that are generally readily available. The phantoms can be easily constructed at minimal cost. This is important for wide distribution of the methodology globally. Of greatest importance is ensuring that the Cu square has clean, precise edges for the MTF determination and that the attenuator is homogenous enough to obtain uniform images in the absence of other disturbing elements. During the pilot survey for methodology verification, MTF data of the mammography devices were very stable. This is in agreement with Rogge and colleagues who studied MTF for 56 digital mammography systems, evaluated during half yearly QC tests.^21^ CV of the MTF values in that study was typically between 0.01 and 0.09. These CVs are in agreement with what has been observed here.

Each individual user should decide which confidence levels have to be used in the longitudinal tracking of the calculated parameters of the different systems. Deciding upon the confidence levels is not a trivial exercise and care is required, especially in the beginning of the implementation period. Using a standard value (e.g., 10 or 15% of baseline) can be a reasonable solution; however, too narrow levels might prompt frequent false alarms, while too wide levels would give a wrong sense of safety and mask poor performance that requires follow‐up. Ideally, the confidence levels are linked to the impact of a deviating parameter on clinical IQ. It is, however, not known what degree change would be acceptable. Large comparative clinical or virtual clinical trials would be required to find the limiting values. In QC practice, very often another philosophy is used: ensure the variations are within confidence limits set by SDs obtained from normal practice. Consequently, a larger amount of initial data of the device itself is required. The Excel spreadsheet developed under the IAEA methodology has built in capability to calculate SDs. Moving averages that adapt to the tendencies in the parameters can also be provided. It is important not just to focus on action limits, but also to observe data for upward or downward trends. An “adoption phase”, during which the results would be more closely monitored by the local staff and the corresponding CQMP along with corrective measures as necessary, would be beneficial.

Trend analysis of all results so far obtained in this pilot survey showed that some X‐ray devices had fluctuations compatible with random errors, while other modalities had issues that were correctly reflected in the data. The major service actions taken during the study (such as replacement of an X‐ray tube and change of a mammography detector) lead to significant modification of IQ metrics values. The number of observations that should have had follow‐up corrective action during this pilot survey from only a small sample of systems was surprisingly high, pointing to the need to apply daily/weekly QC on all imaging modalities. Some of the fluctuations observed derived from personnel rotation in a large hospital. It showed that not all of them were as adept as needed for acquiring the phantom QC images. Training of the local personnel is an essential initial effort that must be undertaken by the supervising CQMP, as cooperation is a key to the success of the program, as it is with any formal QC program in any radiology department. For well‐trained medical radiation technologists, the time invested in QC using the current procedure will remain limited, requiring just a single image on each unit each day or week. Even busy facilities should be able to find time for this activity. They may find communication of the results very motivating.

The novel IAEA phantom and the software methodology were tested on digital systems in a pilot survey with limited number and types of X‐ray radiography and mammography systems. As film‐screen imaging is still common in many parts of the world, it is important to note that the phantom would also be useful for film‐screen systems. From the Cu targets and the surrounding background signal, densitometric evaluations could be performed, and trend analysis is possible. Stability of exposure settings and homogeneity could be verified as well. QC testing in film screen mammography has been recognized to be very beneficial.^2^, ^4^, ^22^ The present test tool allows for easily tracking parameters over time. Trend analysis, carried out by the Excel spreadsheet, is a complementary tool that could also be used with other input parameters. The combined phantom and software procedure come to its full strength, however, on digital systems. The described procedure is consistent with the latest insights on QC, namely to include data from the DICOM header (including the EI) along with IQ metrics that are known to correlate with the clinical tasks that will be performed with the system.^23^ The procedure was tested on both CR and DR systems, and showed, as expected, that there is a difference between CR and DR technologies regarding the stability and the level of some of the IQ metrics.

The ATIA software, which automatically analyzes the images produced by the phantoms, is straightforward and intuitive. The IAEA methodology could be used to improve performance of X‐ray systems as part of a successful radiation‐dose optimization process.

The mammographic phantom that was designed and implemented in this project might appear to be redundant in many parts of the world where the modality already undergoes significant testing. Many of these tests, however, are subjective whereas the evaluation using the IAEA methodology is objective. The ATIA software could be used standalone and the phantom could also be adjusted to be an adjunct to national regulations. In Europe, the European Guidelines on physicotechnical control in film screen and later digital mammography suggested that the quality of breast cancer screening modalities should be centrally controlled by CQMP or medical physics experts. Where this has not yet been put in place, the new IAEA phantom and software could be used. In one study, daily QC results are given from 107 systems tracked daily over a period of 5 years with a simple homogeneous phantom and DICOM header information.^24^ A total of 259 issues have been detected in the daily QC, notwithstanding extensive, mandatory half yearly tests. The majority of the problems had to do with localized “pixel” artifacts (128/259) and either dead lines or columns (28/259), all of these analyzed from the variance map. While the direct impact of daily QC on the screening results cannot be calculated, daily QC was considered worth the effort as many problems could be found and fixed before they would impact clinical quality. With a well‐automated procedure, a single CQMP could oversee a large number of modalities or even facilities remotely. This is especially relevant if access to remote parts of the country is prohibitive.

The study suffered from several weaknesses. The approach of not addressing issues that were identified led to some of the data being questionable. Further, while major events were captured in the study, such as the X‐ray tube change, more subtle drifts and trends did not occur. Such trends would have been helpful to demonstrate the sensitivity of the methodology. The IAEA has embarked on a larger study involving more institutions worldwide, which will provide significantly more data and potentially more situations that will test its sensitivity. In this preliminary proof‐of‐concept work, dependency on processing algorithm, if for processing images are not available, was not investigated. This also could be a part of the wider study being undertaken.

## 5 CONCLUSIONS

The IAEA methodology presented gives the opportunity for a single CQMP to provide QC services even to remote sites where access is prohibitive. The methodology proposed is feasible and easy to implement. Engineering diagrams of both phantoms are freely available as well as the ATIA software and Excel control charts for global use. The phantoms are easily fabricated and consist of materials that are readily available. The ATIA software is robust and reproducible and the implemented metrics provide the latest objective measures of IQ, making them also a valuable adjunct to traditional QC approaches. Automated and remote QC can now be performed on units for which QC has been lacking. This program can be undertaken using various scenarios, depending on the personnel and technology available to the health facility. At its most basic, the facility can analyze the images locally and transmit just the results to the CQMP. On the other end of the spectrum, if infrastructure allows, images can be uploaded to a centralized library where the analysis and logging could be completed automatically with automated alerts sent to the CQMP. If QC testing is performed by a medical radiation technologist, training is essential to ensure accurate image acquisition consistently. At implementation, it is necessary to establish baseline values and ensure that the image, including the DICOM header, is properly populated and transmitted. This program will allow a CQMP to supervise and coordinate the QC/QA program of many remote sites. Regular, daily, or weekly QC of mammographic equipment is known to result in decreased radiation exposure and improved IQ. It is anticipated that regular QC program for radiography and mammography, when implemented on a large scale worldwide, will have a similar impact.

## AUTHOR CONTRIBUTION

All listed authors contributed to this work as detailed below:

Mora: Concept development, data collection, and manuscript preparation.

Pfeiffer: Concept development, data collection, and manuscript preparation.

Zhang: Analysis of software.

Bosmans: Concept development and manuscript preparation.

Delis: Concept development and manuscript preparation.

Razi: Data collection and manuscript preparation.

Arreola: Concept development and data collection.

Tsapaki: Project oversight and manuscript preparation.

## CONFLICT OF INTEREST

The authors declare no conflict of interest.

## DATA VALUE STATEMENT

Author elects to not share data.

## Supporting information

Supporting Information
