# Supplementary material for: The IAEA remote and automated quality control methodology for radiography and mammography
Source: J Appl Clin Med Phys. 2021 Oct 8;22(11):126–42. doi: 10.1002/acm2.13431 (PMC8598138; doi:10.1002/acm2.13431)
Supplement: Supplementary file 1 — Supporting Information [file ACM2-22-126-s001.docx]

**The IAEA remote and automated quality control methodology for radiography and mammography**

Patricia Mora^1^, Douglas Pfeiffer^2^, Gouzhi Zhang^3^, Hilde Bosmans^3^, Harry Delis^4^; Zahra Razi^5^, Manuel Arreola^5^, and Virginia Tsapaki^6^

^1^ San José, Costa Rica

^2^ Boulder Community Health, Boulder, Colorado, United States

^3^ University Hospitals of the KU, Leuven, Belgium

^4^ University of Patras, Greece

^5^ University of Florida Gainesville, Florida, United States

^6^ Human Health Division, International Atomic Energy Agency, Austria

**Corresponding author**

Patricia Mora

Pinares, Curridabat

San José, Costa Rica

patriciamoraucr@gmail.com

**Running title**

Remote and Automated QC

**Author Contribution Statement**

All listed authors contributed to this work as detailed below:

Mora: Concept development, data collection, and manuscript preparation

Pfeiffer: Concept development, data collection, and manuscript preparation

Zhang: Analysis software

Bosmans: Concept development and manuscript preparation

Delis: Concept development and manuscript preparation

Razi: Data collection and manuscript preparation

Arreola: Concept development and data collection

Tsapaki: Project oversight and manuscript preparation

**Acknowledgements**

The data and information presented in the paper is part of an IAEA publication on “Implementation of a remote and automated quality control program for radiography and mammography equipment”.
